# Discordant associations of lipid parameters with albuminuria and chronic kidney disease: a population-based study

**DOI:** 10.1186/s12944-015-0153-8

**Published:** 2015-11-25

**Authors:** Kan Sun, Diaozhu Lin, Feng Li, Chulin Huang, Yiqin Qi, Shengneng Xue, Juying Tang, Chuan Yang, Yan Li, Meng Ren, Li Yan

**Affiliations:** Department of Endocrinology, Sun Yat-sen Memorial Hospital, Sun Yat-sen University, 107 Yanjiang West Road, Guangzhou, 510120 People’s Republic of China

**Keywords:** Dyslipidemia, Low-grade albuminuria, Increased urinary albumin excretion, Chronic kidney disease, TG to HDL-C ratio

## Abstract

**Background:**

Although dyslipidemia is related to the pathogenesis of renal insufficiency, which routinely available lipid measure is more applicable in estimation of kidney function is still uncertain. Our objective was to evaluate inconsistent associations of lipid profiles with both albuminuria and chronic kidney disease (CKD).

**Methods:**

We performed a population-based study in 9730 subjects aged 40 years or older. Definitions of abnormalities in albumin excretion were according to the latest guidelines of American Diabetes Association’s Standards of Medical Care. CKD was defined as estimated glomerular filtration rate (eGFR) < 60 mL/min per 1.73 m^2^ or the presence of albuminuria.

**Results:**

There were 2274 (23.4 %) participants categorized as low-grade albuminuria, 639 (6.6 %) participants categorized as increased urinary albumin excretion and 689 (7.1 %) participants categorized as CKD. Triglycerides (TG), high-density lipoprotein cholesterol (HDL-C), Non HDL-C to HDL-C ratio, TG to HDL-C ratio were significantly correlated with urinary albumin to creatinine ratio (ACR), serum creatinine and eGFR (all *P* < 0.0001). Compare with other lipid parameters, TG to HDL-C ratio have shown the strongest correlation with increased odds of both increased urinary albumin excretion and CKD. No significant associations between lipid parameters and low-grade albuminuria were observed after adjustments for potential confounding factors.

**Conclusion:**

Our study lends support to discordant associations of lipid parameters with albuminuria and renal function. TG to HDL-C ratio is a better marker than other routine lipid measures for identifying renal insufficiency and should be given more consideration in the clinical practice.

## Background

In recent years, chronic kidney disease (CKD) has become an important public health problem and directly affects the global burden of death caused by cardiovascular diseases (CVD) [1, 2]. Increased urinary albumin excretion is including historical micro- and macro-albuminuria, which is defined as the urinary albumin-to-creatinine ratio (ACR) ranges greater or equal than 30 mg/g [3]. Studies conducted over the past decades have provided substantial evidence that increased urinary albumin excretion is a risk factor for diabetic nephropathy and cardiovascular diseases [4–6]. Recent studies indicated that low-grade albuminuria (ACR less than 30 mg/g) is associated with abnormal cardiac mechanics and might also increase the risk of cardiovascular morbidity and mortality [7–9].

As a modifiable risk factors for cardiovascular disease, controlling of dyslipidemia is one of the most effective way for preventing atherosclerotic cardiovascular disease (ASCVD) in subject with chronic renal insufficiency [10–12]. Atherosclerotic renovascular disease (ARVD) is a fairly common disorder in elderly people [13]. However, based on current evidences, the association between dyslipidemia and ARVD is still not confirmed [14, 15]. There is ongoing debate regarding the importance of lipoprotein subclasses and other related markers of lipoprotein metabolism. Guidelines for the detection and treatment of high cholesterol recommend using low density lipoprotein cholesterol (LDL-C) as the primary marker to guide therapy. In addition to LDL-C, Non-high density lipoprotein cholesterol (Non-HDL-C), Non-HDL-C to HDL-C ratio (Non-HDL-C/HDL-C) and triglyceride to HDL-C ratio (TG/HDL-C) are routinely available from the standard lipid profiles. Growing evidences suggests that Non-HDL-C may provide a more accurate measure of coronary heart disease risk than LDL-C [16]. However, in patients with type 2 diabetes, Non-HDL-C/HDL-C is better than Non-HDL-C to predict coronary heart disease [17]. Based on the above arguments, as ASCVD, ARVD and CKD sharing similar pathogenesis and common risk factors, we speculated that the inconsistent association of lipid parameters with vascular might also existed in renal insufficiency.

Previously existing literature and primary analyses have not been implemented to make a systematic comparison on the association of different lipid markers with both of the albuminuria and CKD. In practice, however, it is unclear which routinely available lipid measure is better for the identification of kidney function. Accordingly, we assessed the associations of all routine lipid measures with both albuminuria and renal function in a community-based population.

## Methods

### Study population and design

We performed a cross-sectional study in a community in Guangzhou, China from June to November, 2011. The study population was from the Risk Evaluation of cAncers in Chinese diabeTic Individuals: A lONgitudinal (REACTION) study, which was been set up as a multicenter prospective observational study with the aim of evaluating chronic diseases in the Chinese population [18, 19]. During the recruiting phase, a total of 10,104 residents aged 40 years or older were invited to participate by examination notices or home visits. In total, 9916 subjects signed the consent form and agreed to participate in the survey, and the participation rate was 98.1 %. The subjects who failed to provided information about lipid parameters (HDL-C: *n* = 20; LDL-C: *n* = 1; TG: *n* = 8) or ACR (*n* = 157) were excluded from the analyses. Finally, a total of 9730 eligible individuals were included in the data analyses. The study protocol was approved by the Institutional Review Board of the Sun Yat-sen Memorial Hospital affiliated with Sun Yat-sen University and was in accordance with the principles of the Helsinki Declaration II. Written informed consent was obtained from each participant prior to data collection.

### Clinical and biochemical measurements

A standard questionnaire was administered by trained staff to collect information about sociodemographic characteristics, family history and lifestyle factors. Smoking or drinking habits were classified as ‘never’, ‘current’ (smoking or drinking regularly in the past 6 months) or ‘ever’ (cessation of smoking or drinking of more than 6 months) [20]. A short form of the International Physical Activity Questionnaire (IPAQ) was used to estimate physical activity during leisure time by adding the results for questions about the frequency and duration of moderate or vigorous activities and walking [21]. Metabolic equivalent hours per week (MET-h/week) were calculated separately to evaluate total physical activity.

All participants completed the anthropometrical measurements with the assistance of trained staff using standard protocols [18]. Blood pressure was measured three times consecutively by the same observer with 5 min intervals using an automated electronic device (OMRON, Omron Company, China). The average of the three measurements of blood pressure was used for the analysis. Body height and body weight were recorded to the nearest 0.1 cm and 0.1 kg, respectively, while participants were wearing light indoor clothing without shoes. Body mass indices (BMI) were calculated as the weight in kilograms divided by the height in meters squared (kg/m^2^). Obesity was defined by BMI equal to or greater than 28, and overweight was defined by BMI equal to or greater than 24 and less than 28 [22]. Waist circumference (WC) was measured at the umbilical level with participant in the standing position at the end of a gentle expiration. All relevant data was recorded into the database by 2 different persons to ascertain results reliability in the present study. We performed source data confirmation including verification of all relevant data and informed consent of participating participants. Data queries will be raised for inconsistent, impossible or missing data to ensure that all data are reliable and have been processed correctly.

Venous blood samples were collected for laboratory tests after an overnight fasting of at least 10 h. Measurements of fasting serum insulin, fasting plasma glucose (FPG), oral glucose tolerance test (OGTT) 2 h glucose, triglycerides (TG), total cholesterol (TC), high-density lipoprotein cholesterol (HDL-C), low-density lipoprotein cholesterol (LDL-C) and serum creatinine (SCr) were performed with an autoanalyzer (Beckman CX-7 Biochemical Autoanalyser, Brea, CA, USA). Non-HDL-C levels were calculated from the difference between serum TC and HDL-C. Dyslipidemia was determined if any one of the following indexes was met: TC level ≥ 240 mg/dL, LDL-C level ≥ 160 mg/dL, TG level ≥ 200 mg/dL, HDL-C level < 40 mg/dL or previously diagnosed dyslipidemia [23]. Hemoglobin A1c (HbA1c) was assessed by high-performance liquid chromatography (Bio-Rad, Hercules, CA). The insulin resistance index (homeostasis model assessment of insulin resistance, HOMA-IR) was calculated as fasting insulin (μIU/ml) × fasting glucose (mmol/L)/22.5 [24]. Insulin resistance was defined by a HOMA-IR index within the top quartile (greater than 2.54 in the present study) [25]. Diabetes was diagnosed according to the 1999 World Health Organization diagnostic criteria. The abbreviated Modification of Diet in Renal Disease (MDRD) formula recalibrated for Chinese population was used to calculate estimated glomerular filtration rate (eGFR) expressed in mL/min per 1.73 m^2^ using a formula of eGFR = 186 × [serum creatinine × 0.011]^-1.154^ × [age]^-0.203^ × [0.742 if female] × 1.233, where serum creatinine was expressed as μmol/L and 1.233 was the adjusting coefficient for Chinese population [26].

### Definition of low-grade albuminuria, increased urinary albumin excretion and chronic kidney disease

Definitions of abnormalities in albumin excretion were according to the latest guidelines of American Diabetes Association’s Standards of Medical Care [3]. The first morning spot urine samples were collected for assessing the ACR. Urine albumin and creatinine were measured by chemiluminescence immunoassay (Siemens Immulite 2000, United States) and the Jaffe’ s kinetic method (Biobase-Crystal, Jinan, China) on the automatic analyzer, respectively. ACR was calculated by dividing the urinary albumin concentrations by the urinary creatinine concentrations and expressed in mg/g. Increased urinary albumin excretion was defined according to the ACR ranges greater or equal than 30 mg/g. The definition of low-grade albuminuria was according to the highest quartile of ACR in participants without increased urinary albumin excretion (ACR greater or equal than 11 mg/g and less than 30 mg/g in the present study). Chronic kidney disease (CKD) was defined as eGFR less than 60 mL/min per 1.73 m^2^ or presence of albuminuria (ACR greater or equal than 30 mg/g).

### Statistical analyses

The statistical analyses were performed using SAS version 9.2 (SAS Institute Inc., Cary, NC, USA). Totally, we analyzed the effects of lipid parameters (TG, TC, HDL-C, LDL-C, Non-HDL-C, Non-HDL-C/HDL-C and TG/HDL-C) on clinical factors correlated with renal function (ACR, SCr and eGFR), prevalence of albuminuria (low-grade albuminuria, increased urinary albumin excretion) and CKD.

Continuous variables are presented as the means ± the standard deviations (SD) with the exception of skewed variables, which were presented as medians (interquartile ranges). Categorical variables are expressed as numbers (proportions). FPG, TG, ACR, HOMA-IR, Non-HDL-C/HDL-C, TG/HDL-C, SCr, eGFR and MET-h/week were logarithmically transformed prior to analysis due to non-normal distributions. Linear regression analyses were used to test for trends across groups. Differences between groups were tested with one-way ANOVAs, and *post hoc* comparisons were performed using the Bonferroni correction. Comparisons between categorical variables were performed with the *χ*^2^ test. Pearson’s correlation and multivariate linear regression model were performed to evaluate the associations of lipid parameters with ACR, SCr and eGFR. Unadjusted and multivariate adjusted logistic regression analyses were used to assess the prevalence of low-grade albuminuria, increased urinary albumin excretion and CKD according to elevated lipid profiles quartiles. Participants with CKD were excluded from the analysis for risk of prevalent low-grade albuminuria. The odds ratios (OR) and the corresponding 95 % confidence intervals (95 % CI) were calculated. Model 1 was unadjusted. Model 2 was adjusted for age and sex. Model 3 is further adjusted for BMI, current smoking and drinking status, physical activity level, systolic blood pressure (SBP), and HbA1c. Model 4 is further adjusted for previously diagnosed diabetes, cardiovascular diseases, hypertension and dyslipidemia. The relationships of lipid parameters with increased urinary albumin excretion and CKD were also explored within subgroups that stratified by age (≥55 or < 55 years), degree of obesity (normal, overweight or obesity), hypertension (yes or no), diabetes (yes or no) and dyslipidemia (yes or no). In interaction analyses, we examined separately for feasible associate factors that could modify the relationship between albuminuria and lipid measures. Tests for interaction were performed with including simultaneously each strata factor, lipid parameters quartiles and the respective interaction terms (strata factor multiplied by lipid parameters quartiles) in the final model.

All statistical tests were two-sided, and *P* values < 0.05 were considered statistically significant.

## Results

### Basic clinical characteristics of the study population

Among the 9730 enrolled subjects, the mean age was 55.9 ± 8.1 years and median ACR was 8.1 mg/g with interquartile range 5.7 to 12.2 mg/g. There were 2274 (23.4 %) participants categorized as low-grade albuminuria, 639 (6.6 %) participants categorized as increased urinary albumin excretion and 689 (7.1 %) participants categorized as CKD, respectively. The clinical and biochemical characteristics according to urinary albumin excretion status were presented in Table 1. Compared subjects with normal urinary albumin excretion, those with low-grade albuminuria or increased urinary albumin excretion had significantly higher TG, Non-HDL-C, Non-HDL-C/HDL-C and TG/HDL-C (all *P* < 0.05). Moreover, compared subjects with low-grade albuminuria, those with increased urinary albumin excretion presented with higher TG, Non-HDL-C/HDL-C, TG/HDL-C and lower HDL-C level (all *P* < 0.05).

**Table 1:** Characteristics of study population by urinary albumin excretion status

	Normal urinary albumin excretion	Low-grade albuminuria	Increased urinary albumin excretion	*P* for trend
*n* ( % )	6817 (70.1)	2274 (23.4)	639 (6.6)	
ACR (mg/g)	6.7 (5.0–8.4)	14.7 (12.5–19.0)^*^	53.3 (37.8–94.6)^*#^	<0.0001
Age (years)	55.5 ± 7.7	56.7 ± 8.4^*^	58.0 ± 9.6^*#^	< 0.0001
Male [n (%)]	2097 (30.8)	501 (22.0)	188 (29.4)	< 0.0001
BMI (kg/m^2^)	23.4 ± 3.2	24.0 ± 3.8^*^	24.7 ± 3.7^*#^	< 0.0001
WC (cm)	81.1 ± 9.2	82.3 ± 9.9^*^	85.1 ± 10.4^*#^	< 0.0001
SBP (mmHg)	124.2 ± 15.6	129.0 ± 16.9^*^	135.0 ± 18.9^*#^	< 0.0001
DBP (mmHg)	74.6 ± 9.5	76.4 ± 10.4^*^	78.9 ± 11.0^*^	< 0.0001
Current smoker [*n* (%)]	703 (10.6)	164 (7.4)^*^	74 (11.8) ^#^	0.072
Current drinker [*n* (%)]	226 (3.4)	67 (3.1)	23 (3.7)	0.817
Physical activity (MET-h/week)	21.0 (10.5–45.0)	21.0 (10.5–42.0)	21.5 (10.5–42.0)	0.831
TG (mg/dL)	108.8 (79.6–157.5)	115.9 (82.3–169.9)^*^	137.2 (96.5–194.7)^*#^	< 0.0001
TC (mg/dL)	199.6 ± 47.4	203.6 ± 48.7^*^	202.4 ± 52.0	0.002
HDL-C (mg/dL)	51.2 ± 13.9	51.1 ± 13.8	47.8 ± 12.8^*#^	< 0.0001
LDL-C (mg/dL)	120.8 ± 36.5	122.2 ± 37.8	122.6 ± 39.0	0.067
Non-HDL-C (mg/dl)	148.4 ± 41.8	152.4 ± 43.2^*^	154.6 ± 46.3^*^	< 0.0001
Non-HDL-C/HDL-C	2.9 (2.3–3.6)	3.0 (2.4–3.8)^*^	3.3 (2.6–4.0)^*#^	< 0.0001
TG/HDL-C	2.2 (1.5–3.5)	2.3 (1.5–3.7)^*^	3.0 (1.9–4.5)^*#^	< 0.0001
FPG (mmol/L)	5.4 (5.0–5.9)	5.5 (5.0–6.1)^*^	5.6 (5.1–6.7)^*#^	< 0.0001
HbA1C (%)	6.0 ± 0.7	6.2 ± 1.0^*^	6.5 ± 1.5^*#^	< 0.0001
HOMA-IR	1.67 (1.19–2.39)	1.88 (1.29–2.80)^*^	2.23 (1.45–3.48)^*#^	< 0.0001
SCr (μmol/L)	67.0 (60.8–76.0)	65.0 (59.5–72.9)^*^	69.1 (60.9–81.1)^*#^	0.531
eGFR (ml/min per 1.73 m^2^)	110.9 (100.0–123.8)	111.5 (100.1–124.8)	106.4 (94.2–122.0)^*#^	< 0.0001
Previous CVD (%)	205 (3.0)	78 (3.4)	35 (5.5)^*#^	0.003
Previous hypertension (%)	893 (13.1)	486 (21.4)^*^	198 (31.0)^*#^	< 0.0001
Previous diabetes (%)	313 (4.6)	201 (8.8)^*^	90 (14.1)^*#^	< 0.0001
Previous dyslipidemia (%)	325 (4.8)	124 (5.5)	49 (7.7)^*#^	0.002

1. Data were means ± SD or medians (interquartile ranges) for skewed variables or numbers (proportions) for categorical variables.

2. *P* for trend was calculated for the linear regression analysis tests across the groups. *P* values were for the ANOVA or χ2 analyses across the groups.

3. ^*^P < 0.05 compared with normal urinary albumin excretion group; ^#^P < 0.05 compared with low-grade albuminuria group.

4. ACR, urinary albumin to creatinine ratio; BMI, body mass index; WC, waist circumference; SBP, systolic blood pressure; DBP, diastolic blood pressure; TG, triglycerides; TC, total cholesterol; HDL-C, high-density lipoprotein cholesterol; LDL-C, low-density lipoprotein cholesterol; FPG, fasting plasma glucose; HOMA-IR, homeostasis model assessment of insulin resistance; SCr, serum creatinine; eGFR, estimated glomerular filtration rate; CVD, cardiovascular diseases.

### Associations between lipid parameters and clinical factors correlated with renal function

As shown in Table 2, Pearson’s correlation analysis revealed that TG, HDL-C, Non-HDL-C/HDL-C, TG/HDL-C were significantly correlated with ACR, SCr and eGFR (all *P* < 0.0001). By performing multivariate linear regression analysis and further adjusted for age and sex, we found that the associations were still persisted (all *P* < 0.0001). However, TC and LDL-C were not correlated with ACR in both Pearson’s correlation and linear regression analysis.

**Table 2:** Pearson’s correlation and multiple regression analysis of lipid parameters associated with ACR, SCr and eGFR

	ACR (mg/g)				SCr (μmol/L)				eGFR (ml/min per 1.73 m^2^)			
	r	*P* value	Standardized β	*P* value	r	*P* value	Standardized β	*P* value	r	*P* value	Standardized β	*P *value
TG (mg/dl)	0.09	< 0.0001	0.09	< 0.0001	0.23	< 0.0001	0.19	< 0.0001	−0.23	< 0.0001	−0.22	< 0.0001
TC (mg/dl)	0.01	0.177	−0.01	0.556	0.25	< 0.0001	0.29	< 0.0001	−0.37	< 0.0001	−0.33	< 0.0001
HDL-C (mg/dl)	−0.05	< 0.0001	−0.07	< 0.0001	0.05	< 0.0001	0.19	< 0.0001	−0.21	< 0.0001	−0.23	< 0.0001
LDL-C (mg/dl)	0.002	0.846	−0.01	0.172	0.20	< 0.0001	0.22	< 0.0001	−0.29	< 0.0001	−0.26	< 0.0001
Non-HDL-C (mg/dl)	0.03	0.002	0.02	0.123	0.27	< 0.0001	0.26	< 0.0001	−0.32	< 0.0001	−0.31	< 0.0001
Non-HDL-C/HDL-C	0.06	< 0.0001	0.07	< 0.0001	0.20	< 0.0001	0.08	< 0.0001	−0.13	< 0.0001	−0.10	< 0.0001
TG/HDL-C	0.09	< 0.0001	0.10	< 0.0001	0.16	< 0.0001	0.06	< 0.0001	−0.09	< 0.0001	−0.06	< 0.0001

1. ACR, urinary albumin to creatinine ratio; TG, triglycerides; TC, total cholesterol; HDL-C, high-density lipoprotein cholesterol; LDL-C, low-density lipoprotein cholesterol; SCr, serum creatinine; eGFR, estimated glomerular filtration rate.

2. ACR, SCr, eGFR, TG, Non-HDL-C/HDL-C and TG/HDL-C levels were logarithmically transformed to achieve a normal distribution.

3. r, correlation coefficient; β, regression coefficient; Multiple regression analysis is adjusted for age and sex.

### Associations between lipid parameters and low-grade albuminuria, increased urinary albumin excretion and CKD

**Fig. 1:**
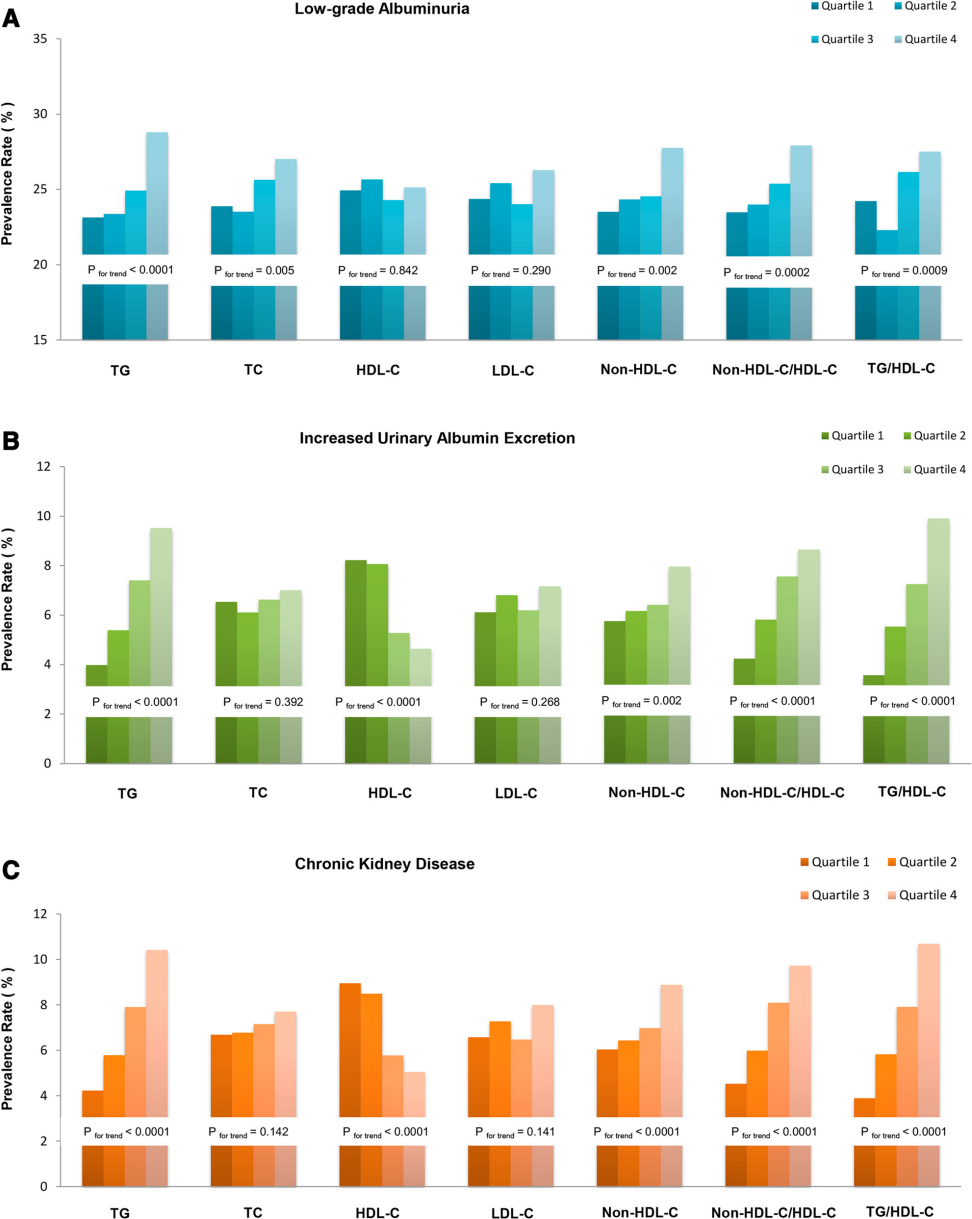
Prevalence of low-grade albuminuria, increased urinary albumin excretion and CKD in quartiles of different lipid profiles

Figure 1 showed the prevalent low-grade albuminuria, increased urinary albumin excretion and CKD in different lipid parameters quartiles. The prevalence of albuminuria and CKD were tended to increase with the elevated TG, Non-HDL-C, Non-HDL-C/HDL-C and TG/HDL-C quartiles (all P for trend < 0.001). For both prevalent increased urinary albumin excretion and CKD, no obvious trend differences were detected in subjects with different quartiles of TC and LDL-C. The prevalence of low-grade albuminuria tended to increase with lipid parameters quartiles except for HDL-C and LDL-C.

As shown in Table 3, in the univariable logistic regression analysis, participants were more likely to have low-grade albuminuria with the elevated quartiles of TG, TC, Non-HDL-C, Non-HDL-C/HDL-C and TG/HDL-C. However, in the multivariable logistic regression, no significant relationships between those lipid parameters and low-grade albuminuria were observed. Similarly in Table 3, in the univariable logistic regression analysis, with the quartile change of HDL-C, Non HDL-C, Non-HDL-C/HDL-C and TG/HDL-C, participants were more likely to have prevalent increased urinary albumin excretion and CKD. Further adjustments for other potential confounding factors including age, sex, BMI, current smoking and drinking status, physical activity level, SBP, HbA1c, previously diagnosed diabetes, cardiovascular diseases, hypertension and dyslipidemia, the associations of those lipid measures with increased urinary albumin excretion and CKD were still persisted.

**Table 3:** Association between lipid parameters and prevalent low-grade albuminuria, increased urinary albumin excretion and CKD

		1-Quartile change of lipid parameters^a^			
		Model 1	Model 2	Model 3	Model 4
Low-grade albuminuria	TG	1.10 (1.06–1.15)	1.10 (1.06–1.15)	1.01 (0.96–1.06)	1.01 (0.97–1.06)
	TC	1.06 (1.02–1.11)	1.03 (0.98–1.07)	1.01 (0.96–1.05)	1.02 (0.97–1.06)
	HDL-C	1.00 (0.96–1.05)	1.05 (1.01–1.10)	0.99 (0.95–1.04)	0.99 (0.94–1.04)
	LDL-C	1.02 (0.98–1.07)	0.99 (0.95–1.04)	0.97 (0.93–1.02)	0.98 (0.94–1.03)
	Non-HDL-C	1.07 (1.03–1.12)	1.05 (1.00–1.09)	1.00 (0.96–1.05)	1.01 (0.96–1.06)
	Non-HDL-C/HDL-C	1.08 (1.04–1.13)	1.10 (1.05–1.15)	1.01 (0.96–1.06)	1.01 (0.96–1.07)
	TG/HDL-C	1.08 (1.03–1.12)	1.09 (1.05–1.14)	0.99 (0.94–1.04)	0.99 (0.94–1.04)
Increased urinary albumin excretion	TG	1.36 (1.27–1.47)	1.34 (1.24–1.44)	1.16 (1.07–1.26)	1.17 (1.08–1.27)
	TC	1.03 (0.96–1.11)	1.01 (0.94–1.08)	0.95 (0.88–1.02)	0.96 (0.89–1.04)
	HDL-C	1.25 (1.16–1.35)	1.26 (1.17–1.36)	1.17 (1.08–1.27)	1.16 (1.07–1.26)
	LDL-C	1.04 (0.97–1.12)	1.02 (0.95–1.09)	0.96 (0.89–1.04)	0.98 (0.91–1.06)
	Non-HDL-C	1.12 (1.04–1.20)	1.09 (1.01–1.17)	0.99 (0.91–1.07)	1.00 (0.93–1.08)
	Non-HDL-C/HDL-C	1.28 (1.19–1.38)	1.25 (1.16–1.35)	1.09 (1.00–1.18)	1.10 (1.01–1.19)
	TG/HDL-C	1.42 (1.32–1.53)	1.40 (1.30–1.51)	1.21 (1.11–1.31)	1.21 (1.11–1.31)
CKD	TG	1.38 (1.29–1.48)	1.35 (1.25–1.45)	1.18 (1.09–1.28)	1.19 (1.10–1.28)
	TC	1.05 (0.98–1.13)	1.03 (0.96–1.10)	0.97 (0.90–1.05)	0.99 (0.92–1.07)
	HDL-C	1.25 (1.16–1.34)	1.25 (1.16–1.34)	1.16 (1.07–1.25)	1.15 (1.06–1.24)
	LDL-C	1.05 (0.98–1.13)	1.03 (0.96–1.10)	0.98 (0.91–1.05)	0.99 (0.92–1.07)
	Non-HDL-C	1.15 (1.07–1.23)	1.11 (1.04–1.20)	1.02 (0.94–1.10)	1.03 (0.96–1.11)
	Non-HDL-C/HDL-C	1.32 (1.23–1.41)	1.28 (1.19–1.37)	1.11 (1.03–1.20)	1.12 (1.04–1.22)
	TG/HDL-C	1.42 (1.32–1.53)	1.39 (1.30–1.50)	1.21 (1.11–1.31)	1.21 (1.11–1.31)

Data are odds ratios (95% confidence interval). Participants without low-grade albuminuria, increased urinary albumin excretion or CKD are defined as 0 and with low-grade albuminuria, increased urinary albumin excretion or CKD as 1

Model 1 is unadjusted

Model 2 is adjusted for age and sex

Model 3 is further adjusted for BMI, current smoking and drinking status, physical activity level, SBP and HbA1c

Model 4 is further adjusted for previously diagnosed diabetes, cardiovascular diseases, hypertension and dyslipidemia

^a^All variables were calculated for 1-Quartile increasing of lipid parameters except for HDL-C, which was calculated for 1-Quartile decreasing

**Fig. 2:**
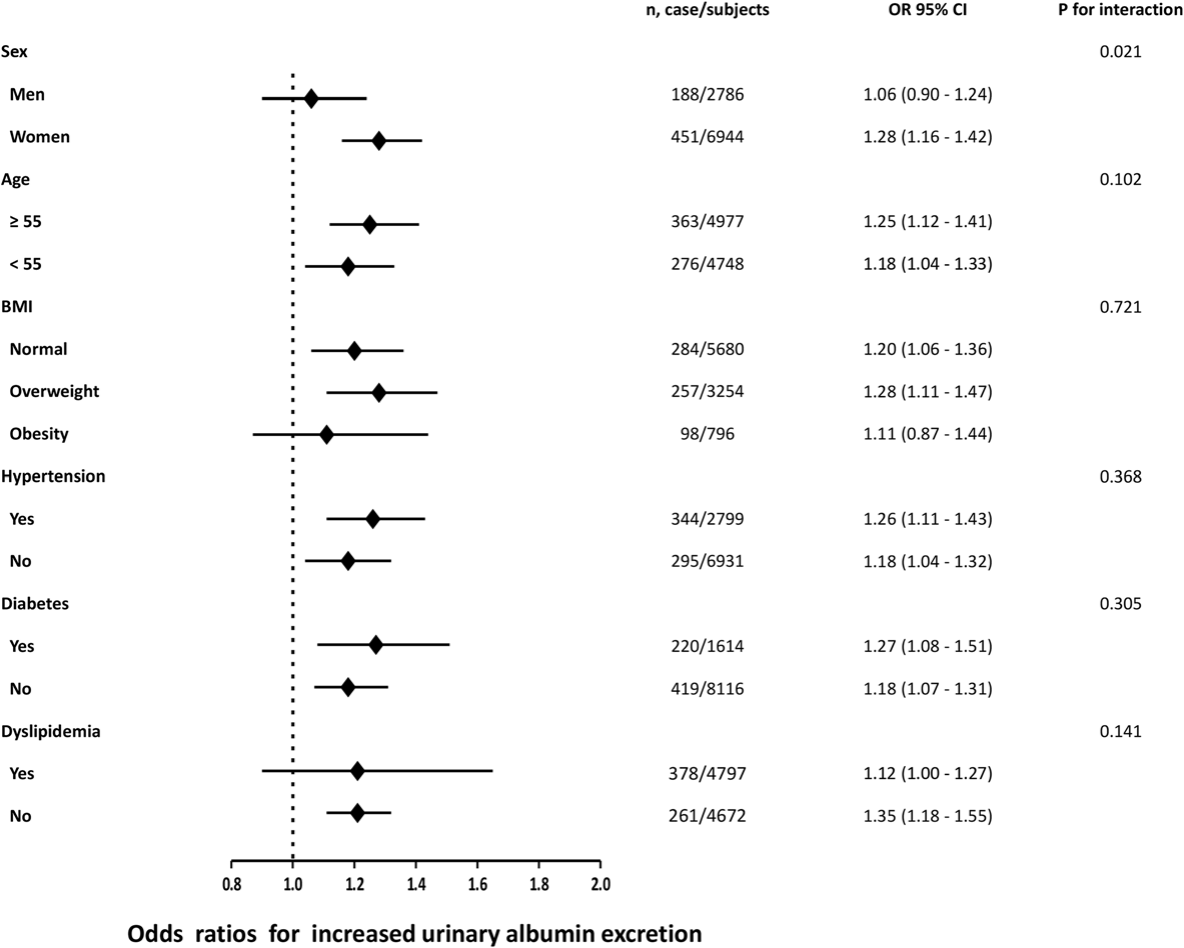
Risk of prevalent increased urinary albumin excretion with each quartile increase of TG/HDL-C level in different subgroups

**Fig. 3:**
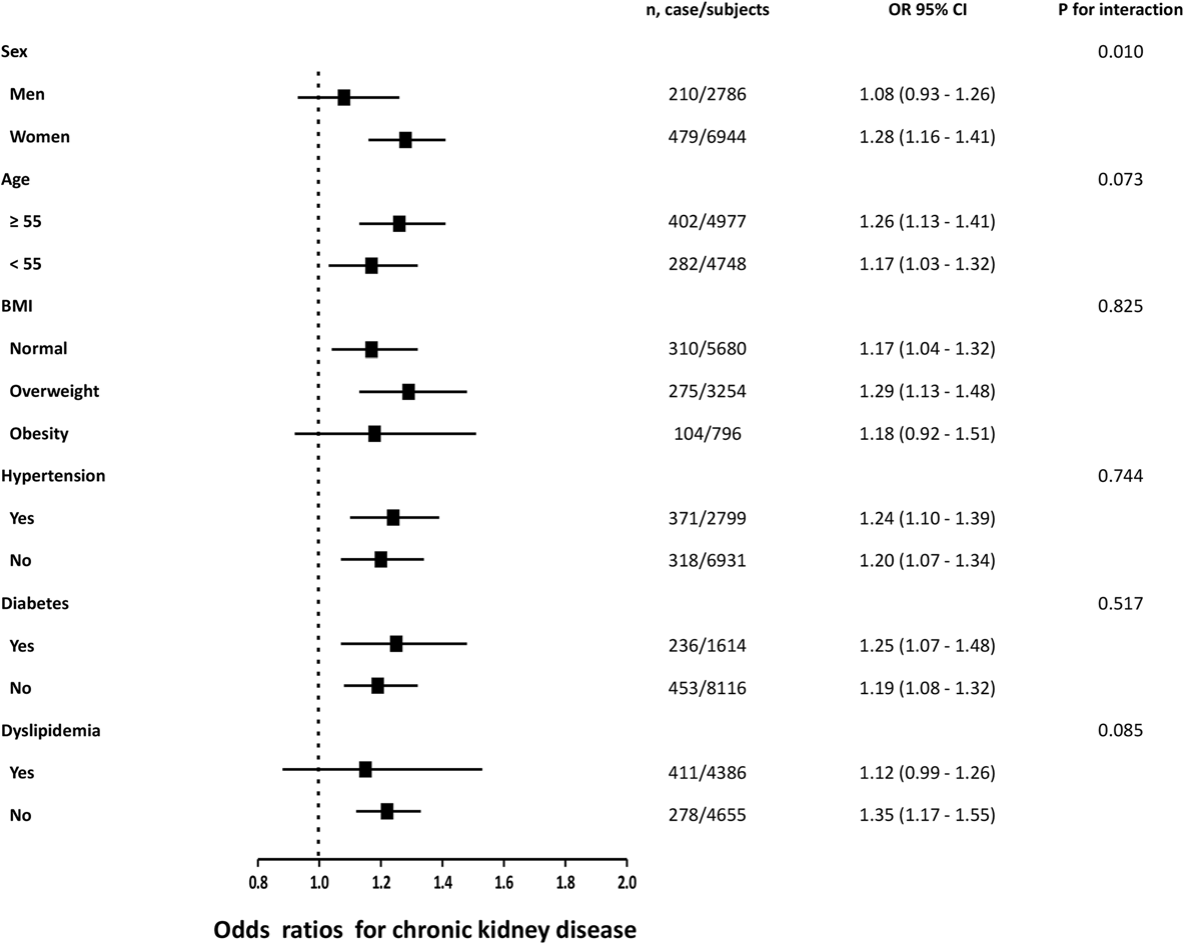
Risk of prevalent CKD with each quartile increase of TG/HDL-C level in different subgroups

Compare with other lipid parameters, TG/HDL-C have shown the strongest correlation with increased odds of both increased urinary albumin excretion and CKD across all logistic regression models (Table 3). To verify the stability of such results, we conducted stratified analyses to determine the odds of increased urinary albumin excretion and CKD with each quartile increase of TG/HDL-C in different subgroups. As shown in Figs. 2 and 3, such associations were not consistently the same according to stratified factors. The associations of TG/HDL-C with prevalent increased urinary albumin excretion and CKD were significant in women strata, both age strata (≥55 and < 55), BMI strata (normal and overweight), both hypertension strata (yes and no), both diabetes strata (yes and no) and subjects without dyslipidemia strata. The difference in the subgroups analysis was accompanied by a statistically significant interaction term when stratified by sex (*P* = 0.021 for increased urinary albumin excretion and *P* = 0.010 for CKD).

## Discussion

In this study of 9730 middle-aged and elderly Chinese, we found significant discordant associations of lipid parameters with albuminuria and CKD. TG/HDL-C is a better marker than other routine lipid measures for identifying both increased urinary albumin excretion and CKD, which should be given more consideration in the clinical approach to risk reduction among those patients. Our data do not support the association between routinely available lipid measures and prevalent low-grade albuminuria.

Lipoprotein abnormalities have been identified as possible causes of renal function damage. Previous study has shown that elevated TG levels and diminished HDL-C levels, but not LDL-C, were associated with increased risk of renal dysfunction and poor renal outcomes [27]. Significant associations between the TG/HDL-C and reduced eGFR or albuminuria have also been identified recently in Korean and Japanese adults [28–30]. In addition to the above lipid measures, a recent study also found that Non-HDL-C/HDL-C is an independent risk factor for the development of CKD [31]. Systemically clarified correlations of different lipid measures with albuminuria and CKD would probably shed light on the prevention and treatment of the diseases. Additionally, growing evidence indicated that low-grade albuminuria is associated with impaired kidney function and incident cardiovascular diseases [32, 33]. Less is known concerning the association between lipid profiles and low-grade albuminuria. In this study, compared with other lipid measures, TG/HDL-C has shown the most significant association with increased urinary albumin excretion and CKD after adjustment for demographic and clinical information. However, no association of lipid measures with low-grade albuminuria was detected after adjustment for same variables.

There are several reasons why the level of TG/HDL-C may be superior to that of other lipid parameters in increased urinary albumin excretion and CKD identification. Elevated TG levels and decreased HDL-C levels have been most strongly associated with increased risk of renal dysfunction [27, 34]. Despite divergent underlying mechanisms, injury-induced cellular TG accumulation can disrupt and damage cellular homeostasis. According to the severity of the injury, TG accumulation could result in renal tubular cell damage in vivo and in vitro models [35]. As a medium of interaction with cell surface proteins, HDL-C could promote TC clearance from the circulation and decrease lipid deposition in the arterial wall, which might slow the progression of kidney dysfunction in patients with CKD [34, 36]. The nature of TG to HDL-C ratio seems to be better correlated with the development of CKD and albuminuria than single measurement of either TG or HDL-C levels. Moreover, insulin resistance is associated with albuminuria and the development of CKD in observational human studies [37]. Compared with other metabolic markers, TG/HDL-C in circulation is better correlated with insulin resistance, which might therefore relevant with risk of various adverse outcomes including renal dysfunction [38]. Based on the results of the present study, the association of TG/HDL-C with increased urinary albumin excretion and CKD was stronger in participants without dyslipidemia. It is thus possible that TG/HDL-C could represent the progression of renal insufficiency even in the early stage of lipid metabolism abnormity.

Several limitations in this study require consideration. First, we evaluated the urinary albumin excretion on a spot morning urine sample. We admitted that multiple samples would provide more stable results for albumin excretion [39]. However, results of spot urine samples correlate well with those of 24 h or multiple urine samples [40, 41]. Use of spot samples for assessing urinary ACR is therefore recommended as a reliable alternative to perform in the out-patient clinic and large epidemiological specimen collection. Second, although it is possible that lipid measures is related with different degrees of albuminuria after controlled for extensive confounding factors, other unmeasured confounders, such as cystatin-C, should be also considered to evaluate to strength the findings of the present study. Third, growing evidence has suggested that abnormal lipid metabolism could contribute to the deterioration of renal function [27, 42]. However, due to the cross-sectional design of the present study, we should be cautious regarding the interpretation of whether dyslipidemia is a causal factor of or a consequence of albuminuria and CKD. Fourth, medications with angiotensin-converting enzyme inhibitors or angiotensin receptor blockers should have affected urinary albumin excretion and should be taken into account when analyzing possible risk factors associated to proteinuria. Absence of these data may influence risk estimates and result interpreting in this setting. Fifth, the results from the present study of Chinese population might not be representative of other races and younger people. The study population was predominantly female, partially because we invited residents with the mean age of 56 years and females are predominant in this age range in China.

## Conclusion

In conclusion, the present study demonstrates discordant associations of lipid parameters with renal insufficiency and TG/HDL-C is a better marker for evaluating increased urinary albumin excretion and CKD. No statistically significant association between lipid parameters and prevalent low-grade albuminuria was found in our data.

## Ethics, consent and permissions

The study protocol was approved by the Institutional Review Board of the Sun Yat-sen Memorial Hospital affiliated with Sun Yat-sen University and was in accordance with the principles of the Helsinki Declaration II. Written informed consent was obtained from each participant prior to data collection.

## Consent to publish

We have obtained consent to publish from the participant to report individual patient data.

## Abbreviations

ACR: Urinary albumin to creatinine ratio
BMI: Body-mass index
CI: Confidence intervals
CVD: Cardiovascular diseases
DBP: Diastolic blood pressure
eGFR: Estimated glomerular filtration rate
FPG: Fasting plasma glucose
HbA1c: Hemoglobin A1c
TG: Triglycerides
TC: total cholesterol
HDL-C: High-density lipoprotein cholesterol
LDL-C: Low-density lipoprotein cholesterol
HOMA-IR: Homeostasis model assessment of insulin resistance
MET-h/week: Metabolic equivalent hours per week
OR: Odds ratios
SBP: Systolic blood pressure
SCr: Serum creatinine
SD: Standard deviation
WC: Waist circumference
